# Supplementary material for: SARS-CoV-2 vaccination elicits broad and potent antibody effector functions to variants of concern in vulnerable populations
Source: Nat Commun. 2023 Aug 24;14:5171. doi: 10.1038/s41467-023-40960-0 (PMC10449910; doi:10.1038/s41467-023-40960-0)
Supplement: Supplementary file 1 — Supplementary Information [file 41467_2023_40960_MOESM1_ESM.pdf]

Supplemental Materials

**Supplemental Figures**	
Supplemental Figure 1	Fc Array data for SARS CoV-2 and VOC S and RBD.
Supplemental Figure 2	Effector functions observed for antibodies specific to SARS-CoV-2 VOC in serum from naïve individuals.
Supplemental Figure 3	IgG antibody responses to OC43 and HKU1 antigens.
Supplemental Figure 4	Immunoglobulin isotypes and antibody effector functions specific for SARS-CoV-2 Wuhan Spike in different conformations and for the S2 domain.
Supplemental Figure 5	Days post second vaccine dose or post symptom onset.
Supplemental Figure 6	Sample THP-1 gating strategy for ADCP assay.
Supplemental Figure 7	Serum antibody effector functions specific to endemic coronavirus antigens.
Supplemental Figure 8	ADCC killing assay comparability and gating.
**Supplemental Tables**	
Supplemental Table 1	Cohort characteristics
Supplemental Table 2	Fc Array reagents

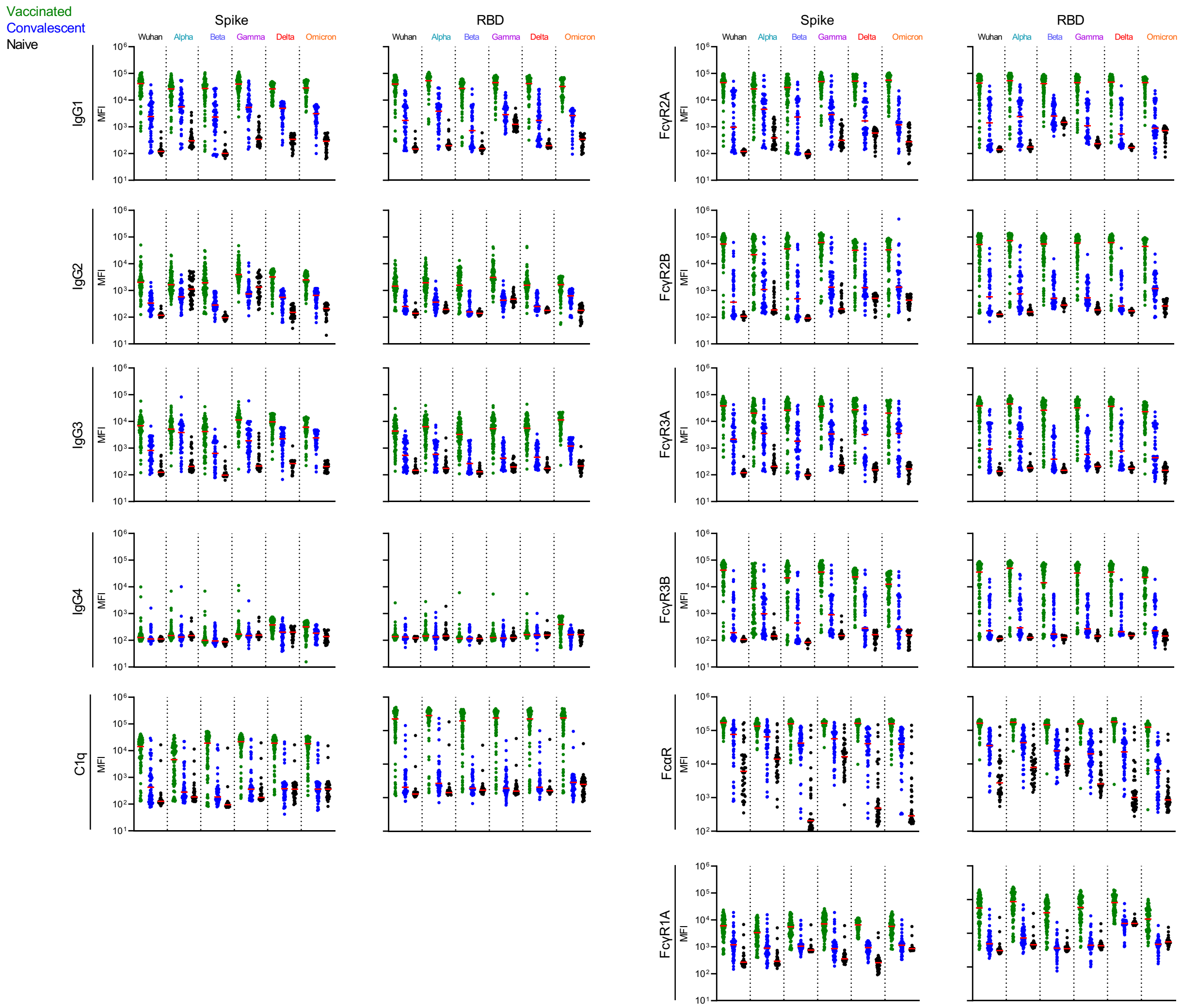

Supplemental Figure 1. Fc Array data for SARS CoV-2 and VOC S and RBD. IgG subclasses and Fc receptor binding activities toward spike and RBD-specific antibodies across Wuhan and VOC proteins in serum from vaccinated (n=87) (green), convalescent (n=57) (blue), and naïve (n=37) (black) subjects. Bar indicates median.

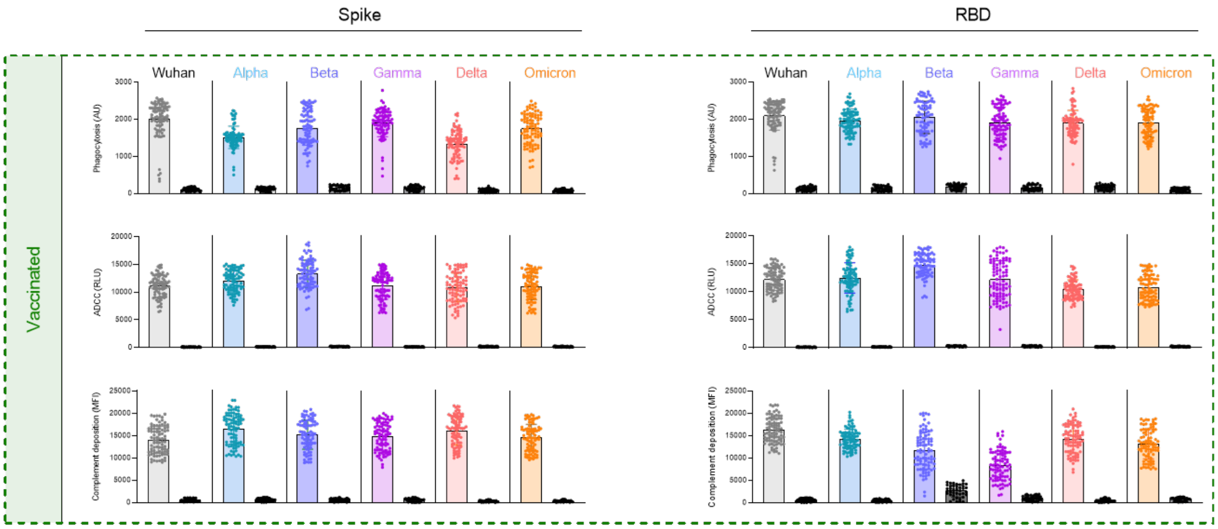

Supplemental Figure 2. Effector function observed for antibodies specific to SARS-CoV-2 VOC in serum from naïve individuals. ADCP (top), ADCC (center), and ADCD (bottom) responses from naïve subjects shown in black for full length spike (left) and RBD (right) SARS-CoV-2 VOC antigens. Responses for naïve (n=37) and vaccinated (n=87) subjects are shown for the 1:50 dilution. Bar indicates median.

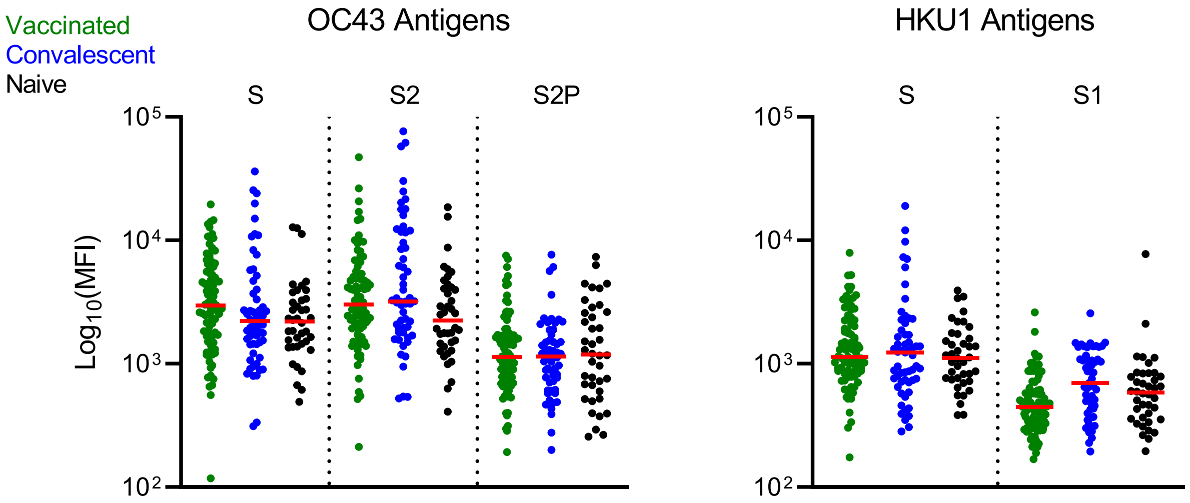

Supplemental Figure 3. IgG antibody responses to OC43 and HKU1 antigens.
Median Fluorescent Intensity (MFI) levels of IgG in serum among vaccinated (n=87) (green), convalescent (n=57) (blue), and naïve (n=37) (black) subjects observed for the full set of OC43 spike protein antigens (left) and HKU1 antigens (right). Bar indicates median.

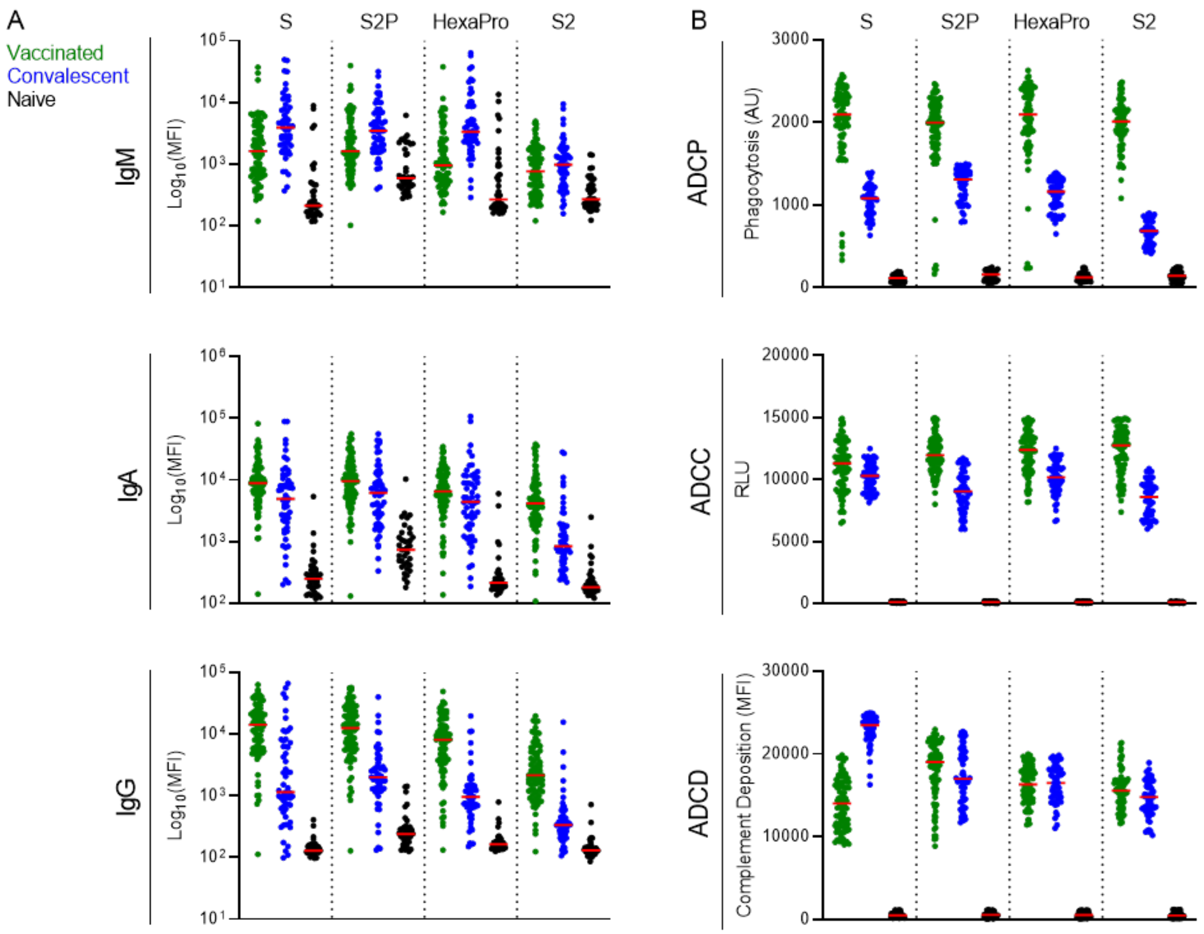

Supplemental Figure 4. Immunoglobulin isotypes and antibody effector functions specific for SARS-CoV-2 Wuhan Spike in different conformations and for the S2 domain. **A**. IgM, IgA, and IgG antibody responses of vaccinated (n=87) (green), convalescent (n=57) (blue), and naïve (n=37) (black) against SARS-CoV-2 Wuhan strain spike domains. **B**. Functional activity of vaccinated (n=87) (green), convalescent (n=57) (blue), and naïve (n=37) (black) serum samples observed against indicated SARS-CoV-2 Wuhan strain spike domains and conformations. Bar indicates median.

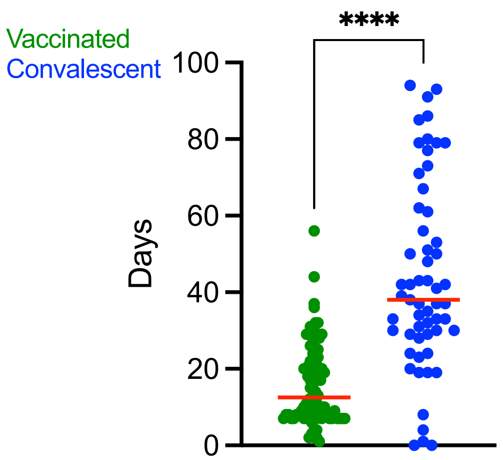

Supplemental Figure 5. Days post second vaccine dose or post symptom onset. Days post second vaccine dose for vaccinated subjects (n=87) (green) and days post SARS-CoV-2 positive PCR result for convalescent subjects (n=57) (blue). Statistical significance was defined by Mann-Whitney test with (****p<0.0001). Bar indicates median.

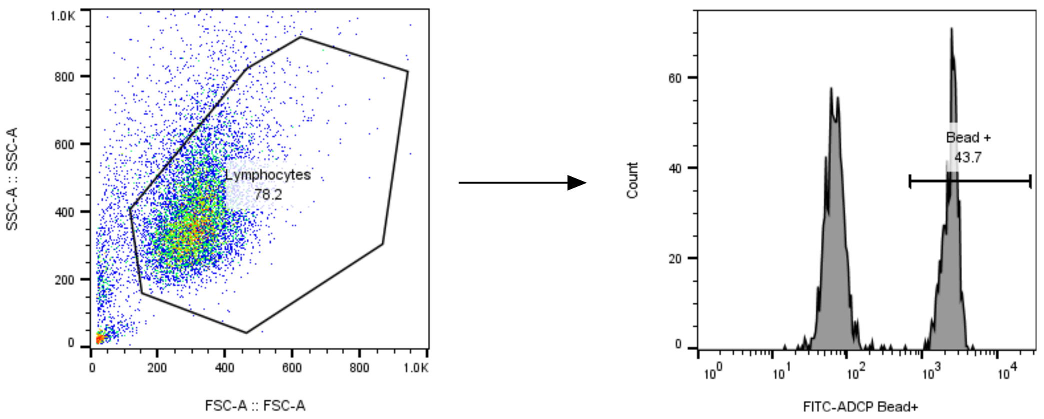

Supplemental Figure 6. Sample THP-1 gating strategy for ADCP assay. Forward and side scatter profile of THP-1 cell gate (left) applied before gating on the bead+ subset (right).

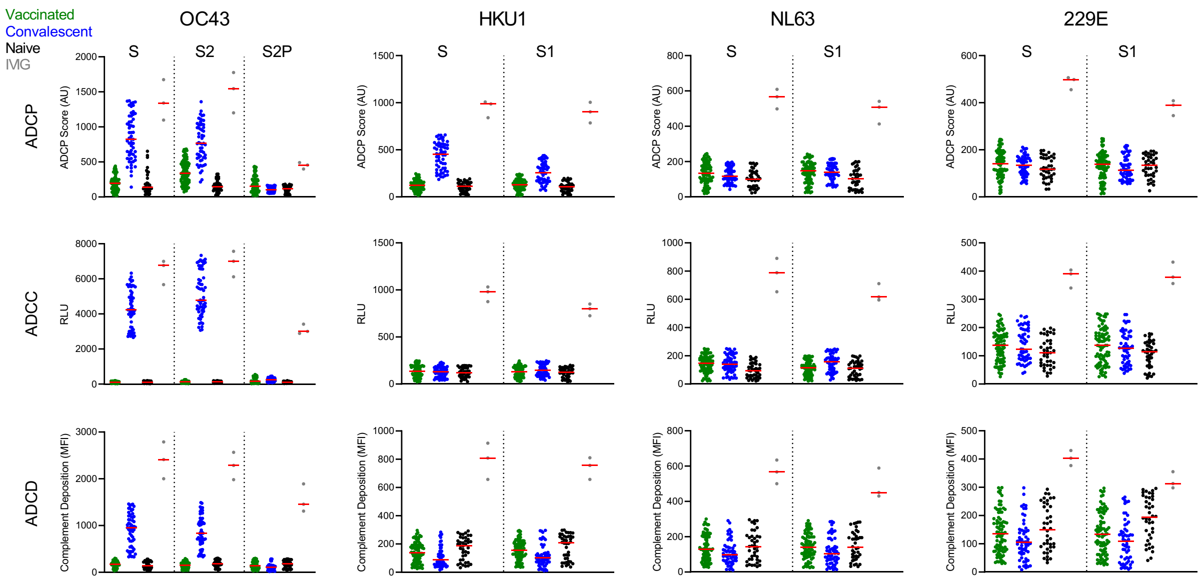

Supplemental Figure 7. Serum antibody effector functions specific to endemic coronavirus antigens. Functional assay data for endemic CoV spike protein variants with high concentration serum-derived IgG (IVIG) presented for comparison for dilute serum from vaccinated (n=87) (green), convalescent (n=57) (blue), and naïve (n=37) (black) subjects. Bar indicates median.

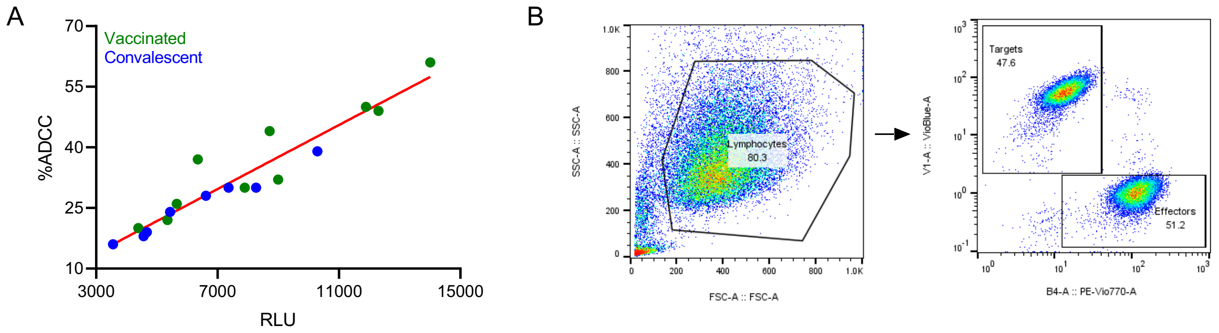

Supplemental Figure 8. ADCC killing assay comparability and gating. A. For a subset of vaccinated (n=10) (green) and convalescent (n=8) (blue) serum samples an NK cell killing assay was performed to determine the correlation between ADCC with a Jurkat reporter cell line (x-axis) and NK cell killing (y-axis)**. B.** Sample gating strategy for NK cell killing assay.

Supplemental Table 1. Cohort characteristics. NA indicates not applicable or available, and IQR indicates interquartile range. Adapted from Crowley, et. al[1].

Characteristic	Convalescent n=19	Pregnant Convalescent n=38	Vaccinated n=37	Pregnant Vaccinated n=50	Naïve controls n=38
Median age (IQR), years	52 (45-62)	31 (27-35)	NA	32 (29-35)	39 (28-50)
Age range (n, %), years	-	-	21-30 (14, 38%)<br>31-40 (8, 22%)<br>41-50 (10, 27%)<br>51-60 (5, 14%)	-	-
Sex (n, %)					
Female	10 (53%)	38 (100%)	17 (46%)	50 (100%)	22 (58%)
Male	9 (47%)	0 (0%)	20 (54%)	0 (0%)	16 (42%)
Median days since PCR+ or symptom onset (IQR)	37 (31-43)	49 (22-78)	NA	NA	NA
Median days since second vaccine dose (IQR)	NA	NA	8 (7-11)	20 (12-29)	NA
Location	US	Belgium	US	Israel	US
IRB	DHMC	CHU St. Pierre	JHMI	Hadassah Medical Center	BioIVT clinical sites
Collection period	March 2020 – April 2020	June 2020 – December 2020	December 2020 - February 2021	February 2021	October 2020
Symptoms or positive test	April 2020 – June 2020	March 2020 – November 2020	NA	NA	NA
Predominant strain	Wuhan	Wuhan	NA	NA	NA

Supplemental Table 2. Fc Array reagents

Antigen	Source	Fc Detection	Source
SARS-CoV-2 S	Acro Biosystems SPN-C82E9	a- IgG	Southern Biotech 2048-09
SARS-CoV-2 S1	Acro Biosystems S1N-C52H3	a-IgG1	Southern Biotech 9054-09
SARS CoV-2 S2-P	Butler et al., 2020[2]	a-IgG2	Southern Biotech 9070-09
SARS CoV-2 S-6P	Hseih, et al, 2020	a-IgG3	Southern Biotech 9210-09
SARS CoV-2 RBD	BEI Resources NR-52366	a-IgG4	Southern Biotech 9200-09
SARS CoV-2 S2	Immune Technology IT-002-034p	a-IgA	Southern Biotech 2050-09
SARS-CoV-2 S Alpha (B.1.1.7)	Sino Biological 40589-V08B6	a-IgM	Southern Biotech 9020-09
SARS-CoV-2 S Beta (B.1.351)	Sino Biological 40589-V08B7	FcγR2aR131	Boesch, et. al, 2014[3]
SARS-CoV-2 S Gamma (P.1)	Sino Biological 40589-V08B8	FcγR2b	Boesch, et al., 2014[3]
SARS-CoV-2 S Delta (B.1.617.2)	Sino Biological 40589-V08B12	FcγR3aV158	Boesch, et al., 2014[3]
SARS-CoV-2 S Omicron (B.1.1.529)	Sino Biological 40589-V08H26	FcγR3bNA2	Boesch, et al., 2014[3]
SARS-CoV-2 RBD Alpha (B.1.1.7)	Sino Biological 40592-V08H82	FcαR	Butler et al., 2020[2]
SARS-CoV-2 RBD Beta (B.1.351)	Sino Biological 40592-V08H4	C1q	Sigma Aldrich C1740
SARS-CoV-2 RBD Gamma (P.1)	Sino Biological 40592-V08H86		
SARS-CoV-2 RBD Delta (B.1.617.2)	Sino Biological 40592-V49H-B		
SARS-CoV-2 RBD Omicron (B.1.1.529)	Sino Biological 40592-V08H121		
SARS-CoV S	Sino Biological 40634-V08B		
SARS-CoV S1	Sino Biological 40634-V08B		
MERS S	Sino Biological 40069-V08B-B		
MERS S1	Sino Biological 40069-V08B1		
OC43 S	Sino Biological 40607-V08B		
OC43 S-2P	Butler et al., 2020[2]		
OC43 S2	Sino Biological 40069-V08B		
229E S	Sino Biological 40605-V08B		

229E S1	Sino Biological 40601-V08H		
HKU1 S	Sino Biological 40606-V08B		
HKU1 S1	Sino Biological 40602-V08H		
NL63 S	Sino Biological 40606-V08B		
NL63 S1	Sino Biological 40604-V08H		

Supplemental References

1. Crowley, A.R., *et al.* Boosting of cross-reactive antibodies to endemic coronaviruses by SARS-CoV-2 infection but not vaccination with stabilized spike. *Elife* **11**(2022).
2. Butler, S.E., *et al.* Distinct Features and Functions of Systemic and Mucosal Humoral Immunity Among SARS-CoV-2 Convalescent Individuals. *Front Immunol* **11**, 618685 (2020).
3. Boesch, A.W., *et al.* Highly parallel characterization of IgG Fc binding interactions. *MAbs* **6**, 915-927 (2014).
