# Supplementary material for: SARS-CoV-2 vaccination elicits broad and potent antibody effector functions to variants of concern in vulnerable populations
Source: Nat Commun. 2023 Aug 24;14:5171. doi: 10.1038/s41467-023-40960-0 (PMC10449910; doi:10.1038/s41467-023-40960-0)
Supplement: Supplementary file 3 — Reporting Summary [file 41467_2023_40960_MOESM3_ESM.pdf]

nature portfolio

Corresponding author(s): Margaret E. Ackerman

Last updated by author(s): Aug 4, 2023

Reporting Summary

Nature Portfolio wishes to improve the reproducibility of the work that we publish. This form provides structure for consistency and transparency in reporting. For further information on Nature Portfolio policies, see our Editorial Policies and the Editorial Policy Checklist.

Statistics

For all statistical analyses, confirm that the following items are present in the figure legend, table legend, main text, or Methods section.

n/a	Confirmed	
☐	☒	The exact sample size (n) for each experimental group/condition, given as a discrete number and unit of measurement
☐	☒	A statement on whether measurements were taken from distinct samples or whether the same sample was measured repeatedly
☐	☒	The statistical test(s) used AND whether they are one- or two-sided<br>*Only common tests should be described solely by name; describe more complex techniques in the Methods section.*
☒	☐	A description of all covariates tested
☐	☒	A description of any assumptions or corrections, such as tests of normality and adjustment for multiple comparisons
☐	☒	A full description of the statistical parameters including central tendency (e.g. means) or other basic estimates (e.g. regression coefficient) AND variation (e.g. standard deviation) or associated estimates of uncertainty (e.g. confidence intervals)
☒	☐	For null hypothesis testing, the test statistic (e.g. F, t, r) with confidence intervals, effect sizes, degrees of freedom and P value noted<br>*Give P values as exact values whenever suitable.*
☒	☐	For Bayesian analysis, information on the choice of priors and Markov chain Monte Carlo settings
☒	☐	For hierarchical and complex designs, identification of the appropriate level for tests and full reporting of outcomes
☐	☒	Estimates of effect sizes (e.g. Cohen's d, Pearson's r), indicating how they were calculated

Our web collection on statistics for biologists contains articles on many of the points above.

Software and code

Policy information about availability of computer code

Data collection	No code was used in data collection
Data analysis	Software used:<br>GraphPad Prism v9<br>FlowJo v10

For manuscripts utilizing custom algorithms or software that are central to the research but not yet described in published literature, software must be made available to editors and reviewers. We strongly encourage code deposition in a community repository (e.g. GitHub). See the Nature Portfolio guidelines for submitting code & software for further information.

Data

Policy information about availability of data

All manuscripts must include a data availability statement. This statement should provide the following information, where applicable:

- Accession codes, unique identifiers, or web links for publicly available datasets
- A description of any restrictions on data availability
- For clinical datasets or third party data, please ensure that the statement adheres to our policy

Data presented in this work is provided as Source Data File 1. Statistical significance of group differences are provided as Source Data File 2.

Human research participants

Policy information about studies involving human research participants and Sex and Gender in Research.

Reporting on sex and gender	Samples were collected from male and female participants in both vaccinated and convalescent groups. Neither sex as a biological variable nor gender were considered in design or analysis.
Population characteristics	Cohort characteristics are available in Supplemental Table 1. Each patient provided serum for our study. Naive patient serum samples were commercially sourced.
Recruitment	Vaccinated subjects were recruited were health care professionals and laboratory workers. They were compensated for blood donations. Convalescent subjects from DHMC were Covid-19 positive subjects from mandatory testing of college students and staff who were approached for enrollment in the study. Pregnant women (vaccinated and convalescent) were admitted to respective hospitals for delivery and eligible for this study based on a positive SARS-CoV-2 test or receipt of an mRNA vaccine. The authors are not aware of further biases in sample collection.
Ethics oversight	Subjects provided informed written consent and studies were reviewed and approved by IRBs at DHMC, JHMI, CHU St. Pierre, and at Hadassah Medical Center, as specified in Supplementary Table 1.

Note that full information on the approval of the study protocol must also be provided in the manuscript.

Field-specific reporting

Please select the one below that is the best fit for your research. If you are not sure, read the appropriate sections before making your selection.

- [x] Life sciences
- [ ] Behavioural & social sciences
- [ ] Ecological, evolutionary & environmental sciences

For a reference copy of the document with all sections, see nature.com/documents/nr-reporting-summary-flat.pdf

Life sciences study design

All studies must disclose on these points even when the disclosure is negative.

Sample size	The study cohort comprised 87 subjects were vaccinated against SARS-CoV-2 with an mRNA vaccine. Of the vaccinated subjects, 50 were pregnant and 37 non-pregnant. Additionally 57 convalescent subjects were included in the study with 38 being pregnant subjects and 19 non-pregnant subjects. 37 naive samples were included as a control group. Sample size was not predetermined due to this being an observational study. Numerous studies have shown differences among antibody responses between vaccinated and convalescent subjects so we believed the sample size would be adequate to see significant differences among groups.
Data exclusions	Three commercially-sourced "naive" samples were excluded based on the observation of SARS-CoV-2 anti-N antibodies.
Replication	Fc array experiments were performed in technical duplicate. Other assays were performed in biological triplicate. Replication experiments were successful.
Randomization	Subjects were not randomized as this was an observational study.
Blinding	Investigators were not blinded to sample groups as they arrived from different sources with distinct labeling. Samples were tested simultaneously and not separated among groups, reducing potential experimental bias.

Reporting for specific materials, systems and methods

We require information from authors about some types of materials, experimental systems and methods used in many studies. Here, indicate whether each material, system or method listed is relevant to your study. If you are not sure if a list item applies to your research, read the appropriate section before selecting a response.

Materials & experimental systems

n/a	Involved in the study	
[ ]	[x]	Antibodies
[ ]	[x]	Eukaryotic cell lines
[x]	[ ]	Palaeontology and archaeology
[x]	[ ]	Animals and other organisms
[x]	[ ]	Clinical data
[x]	[ ]	Dual use research of concern

Methods

n/a	Involved in the study	
[x]	[ ]	ChIP-seq
[ ]	[x]	Flow cytometry
[x]	[ ]	MRI-based neuroimaging

Antibodies

Antibodies used	The following antibodies were used:<br>a- IgG Southern Biotech 2048-09 (1:5000 Dilution)<br>a-IgG1 Southern Biotech 9054-09 (1:1000 Dilution)<br>a-IgG2 Southern Biotech 9070-09 (1:250 Dilution)<br>a-IgG3 Southern Biotech 9210-09 (1:250 Dilution)<br>a-IgG4 Southern Biotech 9200-09 (1:250 Dilution)<br>a-IgA Southern Biotech 2050-09 (1:250 Dilution)<br>a-IgM Southern Biotech 9020-09 (1:250 Dilution)<br>Cell Tracker Orange Invitrogen C3455 (1:1000 Dilution)<br>Cell Tracker Violet Invitrogen C10094 (1:1000 Dilution)<br>Steptavidin-PE Agilent PJ31S-1 (1:5000 Dilution)<br>a-C3b CedarLane CL636AP (1:500 Dilution)<br>anti-mouse IgG1 Southern Biotech 1070-09 (1:1000 Dilution)
Validation	All antibodies have been previously validated in a previous peer reviewed manuscript from our group: PMID 33584712.

Eukaryotic cell lines

Policy information about cell lines and Sex and Gender in Research

Cell line source(s)	THP-1 cells were obtained from ATCC (TIB-202). Jurkat cells were obtained from Invivogen (jktl-nfat-cd16). CEM.NKR cells were obtained from NIH AIDS Reagent program (ARP-4376).
Authentication	Cell lines were authenticated by commercial sources (morphology).
Mycoplasma contamination	Cell lines were not tested for mycoplasma.
Commonly misidentified lines<br>(See ICLAC register)	No cell lines used in this study are commonly misidentified.

Flow Cytometry

Plots

Confirm that:

☒ The axis labels state the marker and fluorochrome used (e.g. CD4-FITC).

☒ The axis scales are clearly visible. Include numbers along axes only for bottom left plot of group (a 'group' is an analysis of identical markers).

☒ All plots are contour plots with outliers or pseudocolor plots.

☒ A numerical value for number of cells or percentage (with statistics) is provided.

Methodology

Sample preparation	Cells were spun down and wash 3 times in FACS buffer before staining. Staining was done for 30 min on ice for all antibodies. After staining cells were wash 3 times and resuspended in FACS buffer prior to running.
Instrument	MACSQuant Analyzer (Miltenyi Biotec)
Software	FlowJo Version 10
Cell population abundance	Viability was measured for cells before analysis and was > 90%.
Gating strategy	FSC and SSC gates were used to identify lymphocytes were were than the population analyzed for ADCP and ADCC assays.

☒ Tick this box to confirm that a figure exemplifying the gating strategy is provided in the Supplementary Information.
